# *Candida* Colonization and Esophagitis in HIV‐Negative Patients: An Endoscopic, Histopathological, and Molecular Study

**DOI:** 10.1155/cjid/2877787

**Published:** 2026-07-21

**Authors:** Fatemeh Nabiei, Rasoul Samimi, Neda Nasirian, Monirsadat Mirzadeh, Milad Badri, Faezeh Mohammadi

**Affiliations:** ^1^ Cellular and Molecular Research Center, Research Institute for Prevention of Non-Communicable Disease, Qazvin University of Medical Sciences, Qazvin, Iran, qums.ac.ir; ^2^ Department of Internal Medicine, School of Medicine, Medical Microbiology Research Center, Velayat Hospital, Qazvin University of Medical Sciences, Qazvin, Iran, qums.ac.ir; ^3^ Department of Pathobiology, School of Medicine, Velayat Hospital, Qazvin University of Medical Sciences, Qazvin, Iran, qums.ac.ir; ^4^ Metabolic Diseases Research Center, Research Institute for Prevention of Non-Communicable Diseases, Qazvin University of Medical Sciences, Qazvin, Iran, qums.ac.ir; ^5^ Medical Microbiology Research Center, Qazvin University of Medical Sciences, Qazvin, Iran, qums.ac.ir; ^6^ Student Research Committee, Qazvin University of Medical Sciences, Qazvin, Iran, qums.ac.ir; ^7^ Department of Medical Parasitology and Mycology School of Medicine, Qazvin University of Medical Sciences, Qazvin, Iran, qums.ac.ir

**Keywords:** *Candida albicans*, *Candida* esophagitis, esophageal biopsy, HIV-negative, non-albicans *Candida*

## Abstract

**Background:**

*Candida* esophagitis (CE) is a recognized opportunistic infection in immunocompromised individuals, but data from HIV‐negative patients remain limited. This study aimed to determine the prevalence, clinical manifestations, risk factors, and molecular characteristics of CE in HIV‐negative adults undergoing upper gastrointestinal endoscopy.

**Methods:**

In this cross‐sectional study, 455 HIV‐negative adults with upper gastrointestinal symptoms who underwent esophagogastroduodenoscopy were evaluated. An esophageal mucosal sample for fungal culture was collected from all patients, while biopsy for histopathology was performed only when endoscopic findings suggested *Candida* infection. Active CE was defined by compatible endoscopic lesions, histopathological evidence of tissue invasion, and a positive fungal culture. *Candida* species were identified using PCR‐RFLP.

**Results:**

*Candida* was detected in 84 patients (18.5%), and histopathologically confirmed that CE was identified in 16 patients (3.5%). The most common symptoms among CE patients were epigastric pain (68.8%) and acid reflux (56.3%). Diabetes mellitus (OR = 6.19, *p* = 0.001), denture use (OR = 3.72, *p* = 0.020), corticosteroid use (OR = 4.54, *p* = 0.025), and antibiotic use (OR = 5.08, *p* = 0.018) were significantly associated with CE. Among patients with CE (*n* = 16), molecular analysis showed *Candida albicans* as the predominant species (81.3%), followed by *C. glabrata* (12.5%) and *C. tropicalis* (6.2%).

**Conclusions:**

CE is an uncommon yet clinically relevant condition in HIV‐negative patients. Metabolic factors, medication exposure, and denture use significantly increase the risk of CE. Early identification of high‐risk individuals and accurate species detection may support timely diagnosis and appropriate management.

## 1. Introduction

*Candida* esophagitis (CE) is one of the most common opportunistic fungal infections and primarily affects immunocompromised individuals, including HIV‐positive patients [1, 2]. In contrast, CE is considered a rare condition in immunocompetent individuals [3]. Although *Candida* species can colonize the esophageal mucosa of healthy people without causing symptoms, infection and inflammation typically develop under specific predisposing factors [3, 4]. Several risk factors have been associated with the development of CE, including advanced age; diabetes mellitus, and malignancies; and the use of antibiotics, corticosteroids, proton pump inhibitors, herbal remedies, and excessive alcohol consumption [5]. Additionally, esophageal motility disorders, particularly achalasia, have been recognized as significant contributors, with a higher prevalence of CE reported among affected patients than in healthy individuals [6]. Clinical manifestations can range from gastrointestinal discomfort to classic esophageal symptoms such as dysphagia or odynophagia [7]. The diagnosis of CE usually relies on endoscopic observation of white plaques and histological confirmation of invasion by yeasts and hyphae into the superficial epithelium [8, 9]. Histopathological examination of biopsy samples provides critical insights into the extent of mucosal involvement, the nature of the inflammatory response, and the presence of fungal elements within the esophageal tissue [2, 10]. While *Candida albicans* is the most frequently implicated species in CE, other *Candida* species can also contribute to the disease [11]. Accurate identification of *Candida* species is essential for the management and effective treatment of esophageal candidiasis [11, 12]. While conventional diagnostic methods such as endoscopy and histopathological examination remain essential, molecular techniques have significantly increased the specificity and sensitivity of *Candida* detection and species differentiation [13, 14]. Molecular identification not only facilitates accurate diagnosis but also provides critical information on epidemiological trends and potential antifungal resistance, which is essential for guiding targeted treatment [15]. This study aimed to determine the prevalence, clinical and histopathological features, associated risk factors, and molecular identification of *Candida* species in HIV‐negative patients undergoing upper gastrointestinal endoscopy.

## 2. Materials and Methods

### 2.1. Study Design and Population

This cross‐sectional study was conducted at Velayat Hospital in Qazvin, Iran, from December 2023 to January 2025. A total of 455 adult outpatients who presented with upper gastrointestinal symptoms and underwent upper endoscopy (EGD) were included. Written informed consent was obtained from all participants. The study protocol was approved by the Ethics Committee of Qazvin University of Medical Sciences (IR.QUMS.REC.1402.100) and was carried out in accordance with the Declaration of Helsinki. The inclusion criteria were age 18 years or older and having one or more upper gastrointestinal symptoms, including dysphagia, odynophagia, heartburn, acid reflux, epigastric pain, nausea, or vomiting. Patients with a positive HIV serology test and those with a recent history of systemic or topical antifungal use were excluded from the study. Demographic and clinical data were collected using a standardized questionnaire.

### 2.2. Endoscopic Evaluation and Sampling

EGD was performed by an experienced gastroenterologist using a standard video endoscope. The esophagus was carefully examined for findings suspicious for CE, including adherent white plaques, white exudates, erythematous or friable mucosa, erosions, and ulcers. From all patients, one esophageal mucosal sample was systematically collected and placed in a sterile container with normal saline for mycological analysis to assess *Candida* colonization. In patients with endoscopic findings suspicious for *Candida* infection, an additional esophageal mucosal biopsy was obtained for histopathological evaluation.

### 2.3. Fungal Culture and Identification

Esophageal mucosal samples were immediately transported in sterile containers containing normal saline to the mycology laboratory. The samples were gently mixed, and a portion of the saline suspension containing the mucosal specimen was directly inoculated onto Sabouraud Dextrose Agar (SDA) supplemented with chloramphenicol (0.05 g/L) using a sterile loop. The inoculated plates were incubated at 25°C–30°C for 48–72 h and examined daily for yeast growth. Suspected *Candida* colonies were subcultured onto CHROMagar Candida (CHROMagar Microbiology, Paris, France) for preliminary identification based on colony coloration. Final species identification was confirmed using molecular methods. All isolates were preserved in 10% glycerol at −20°C for DNA extraction and subsequent molecular analyses.

### 2.4. Histopathological Examination

Biopsy specimens obtained from endoscopically suspected lesions were fixed in 10% neutral‐buffered formalin, processed routinely, and embedded in paraffin. Tissue sections were stained with hematoxylin and eosin (H&E) and periodic acid–Schiff (PAS). Histopathological evidence of tissue invasion was defined as the presence of yeast cells, pseudohyphae, or hyphae penetrating the esophageal epithelium or lamina propria with an associated inflammatory response.

### 2.5. Case Definitions

Patients were classified into three mutually exclusive groups based on endoscopic findings, fungal culture results, and histopathology when available. It should be noted that although clinical symptoms were documented for all patients, they were not used as a diagnostic criterion for active CE. Patients with negative esophageal fungal culture and no endoscopic findings suggestive of CE were classified as having no *Candida*. *Candida* colonization was defined as a positive esophageal culture for *Candida* species without histopathological evidence of tissue invasion. Active CE was defined by the presence of endoscopic lesions suggestive of *Candida* infection together with histopathological confirmation of fungal invasion in esophageal tissue and a positive fungal culture.

### 2.6. Molecular Identification

Genomic DNA was extracted from clinical isolates via a combination of glass beads and phenol:chloroform:isoamyl alcohol (25:24:1) [16]. Briefly, a loopful of fresh yeast colonies was mixed with 0.5‐mm diameter glass beads, 500 μL of lysis buffer (100‐mM Tris‐HCl, pH 8; 10‐mM EDTA; 100‐mM NaCl; 1% sodium dodecyl sulfate (SDS)), and 500 μL of phenol:chloroform:isoamyl alcohol (25:24:1) in a 1.5‐mL microcentrifuge tube. The mixture was vortexed vigorously for 5 min, followed by centrifugation at 12,000 rpm for 10 min. The supernatant was carefully transferred to a new tube, and the DNA was precipitated by adding an equal volume of cold isopropanol. The samples were incubated at −20°C for 30 min and centrifuged again at 12,000 rpm for 15 min.

The resulting DNA pellet was washed twice with cold 70% ethanol, air‐dried at room temperature, and resuspended in 50 μL of double‐distilled water. The extracted DNA samples were stored at −20°C until further use. PCR amplification of the internal transcribed spacer (ITS) region was performed in a 25‐μL reaction mixture containing 12.5 μL of Taq Mix Red 2X, 30 pmol of each primer (ITS1: 5ʹ‐TCC GTA GGT GAA CCT GCG G‐3ʹ and ITS4: 5ʹ‐ TCC TCC GCT TAT TGA TAT GC‐3ʹ) [17], and 1 μL of genomic DNA (∼20 ng). The PCR cycling conditions were as follows: initial denaturation at 94°C for 5 min; 30 cycles of denaturation at 94°C for 30 s, annealing at 55°C for 45 s, and extension at 72°C for 1 min; and a final extension at 72°C for 7 min. PCR products were analyzed via electrophoresis on a 1.5% agarose gel.

### 2.7. Restriction Fragment Length Polymorphism (RFLP) Analysis

The PCR amplification products were subjected to RFLP analysis for species identification. The digestion reactions were performed by incubating 10 μL of the PCR product with 10 units of the *MspI* restriction enzyme (Thermo Fisher Scientific, Lithuania), along with the appropriate reaction buffer and sterile distilled water, in a total volume of 20 μL. The mixture was incubated at 37°C for 2 h to allow complete digestion. The resulting fragments were separated via electrophoresis on a 2% agarose gel.

#### 2.7.1. Statistical Analysis

Statistical analyses were performed using SPSS software Version 26 (IBM Corp., Armonk, NY, USA). Categorical variables were compared using the chi‐square test or Fisher’s exact test, and continuous variables were analyzed using the Mann–Whitney *U* test. For risk factor analysis, patients with active CE were compared with all non‐CE patients (a combined group of No *Candida* and Colonization). Because of the limited number of CE cases, multivariable modeling was not feasible, and therefore univariate odds ratios (ORs) with 95% confidence intervals (CIs) were calculated for each potential risk factor. A *p* value < 0.05 was considered statistically significant.

## 3. Results

### 3.1. Patient Characteristics, *Candida* Prevalence, and Histopathological Findings

**FIGURE 1:**
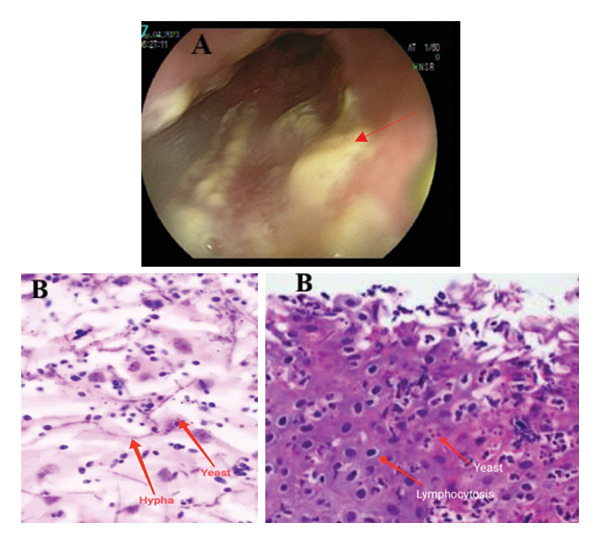
(A) Endoscopic image of esophageal candidiasis showing multiple thick, whitish plaques (red arrow). (B) PAS‐stained esophageal biopsy smear showing both yeast cells and *Candida* (pseudo)hyphae (red arrows) (original magnification × 40).

A total of 455 HIV‐negative patients were enrolled, including 239 females (52.5%) and 216 males (47.5%), with a mean age of 56 ± 16.5 years. Eighty‐four of 455 patients (18.5%) had a positive esophageal *Candida* culture, of whom 16 (3.5% of all patients) had histopathologically confirmed active CE. Histopathological grading (*n* = 16) showed minimal fungal invasion with mild inflammation in 13 patients (81.2%), moderate invasion with moderate inflammation in one patient (6.3%), and severe invasion characterized by abundant yeast, hyphae, and marked inflammatory infiltration in two patients (12.5%) (Figure 1). Moreover, histopathological examination demonstrated that all 16 patients with confirmed CE also exhibited features of chronic esophagitis. No dysplasia or adenocarcinoma was identified, and the histopathological findings were consistent with both culture results and endoscopic observations.

### 3.2. Clinical Symptoms and Associated Risk Factors for CE

In the non‐CE patient group (patients with no *Candida* and those with *Candida* colonization; *n* = 439), the most common clinical symptoms were epigastric pain (*n* = 235; 53.5%), nausea and vomiting (*n* = 149; 34%), and acid reflux (*n* = 118; 27%). Among patients with pathologically confirmed CE (*n* = 16), the most frequently reported symptoms were epigastric pain (*n* = 11; 68.8%) and acid reflux (*n* = 9; 56.3%). Additional clinical symptoms are summarized in Table 1. Among clinical symptoms, acid reflux (OR = 3.49, 95% CI: 1.27–9.60, *p* = 0.019) and heartburn (OR = 3.27, 95% CI: 1.15–9.30, *p* = 0.031) were significantly associated with CE (Table 1). Epigastric pain was the most frequently reported symptom among patients with CE; however, no statistically significant association was observed between epigastric pain and CE (OR = 1.91, 95% CI: 0.65–5.58, *p* = 0.230). Among the patients diagnosed with active CE, 18.75% presented without typical esophageal symptoms. These patients exhibited only minimal fungal invasion and mild inflammation on histopathological examination. The most prevalent patient characteristics in the non‐CE group were antacid use (32%; *n* = 140), denture use (14%; *n* = 61), smoking (13.2%; *n* = 58), and diabetes mellitus and anemia (11.2% each; *n* = 49). Among patients with pathologically confirmed CE (*n* = 16), the most frequent risk factors were diabetes mellitus (*n* = 7; 43.8%), antacid use (*n* = 7; 43.8%), and denture use (*n* = 6; 37.5%) (Table 2). Diabetes mellitus (OR = 6.19, 95% CI: 2.21–17.37; *p* = 0.001), denture use (OR = 3.72, 95% CI: 1.30–10.60; *p* = 0.020), corticosteroid use (OR = 4.54, 95% CI: 1.38–14.95; *p* = 0.025), and antibiotic use (OR = 5.08, 95% CI: 1.53–16.82; *p* = 0.018) were significantly associated with CE (Table 2). Interestingly, antacid use was a frequent characteristic among CE patients; however, it was not statistically associated with CE (*p* = 0.319). All patients with CE had at least one predisposing risk factor. None of the patients with CE reported a history of alcohol use or a history of organ transplantation. There was no significant difference in age between patients with and without CE (*p* = 0.443).

**TABLE 1:** Comparison of clinical symptoms between non‐CE and CE patients.

Variable	Non‐CE patients *n* (%) (*n* = 439)	CE patients *n* (%) (*n* = 16)	OR (95% CI), *p* value
Clinical symptoms			
Epigastric pain	235 (53.5%)	11 (68.8%)	1.91 (0.65–5.58), 0.230
Acid reflux	118 (27%)	9 (56.3%)	3.49 (1.27–9.60), 0.019^∗^
Heartburn	68 (15.5%)	6 (37.5%)	3.27 (1.15–9.30), 0.031^∗^
Nausea and vomiting	149 (34%)	4 (25%)	0.65 (0.21–2.05), 0.457
Dysphagia	21 (4.8%)	2 (12.5%)	2.84 (0.61–13.33), 0.191
Odynophagia	17 (4%)	0 (0%)	NE, 1.000

*Note:* OR, univariate odds ratio; CI, 95% confidence interval. *p* values were calculated using Pearson’s chi‐square test; Fisher’s exact test was used when expected cell counts were small, as appropriate. NE, odds ratio not estimable because no cases occurred in the *Candida esophagitis* group.

^∗^Statistically significant at *p* < 0.05.

**TABLE 2:** Risk factors and their association with *Candida esophagitis* in non‐CE and CE patients.

Risk factor	Non‐CE patients *n* (%) (*n* = 439)	CE patients *n* (%) (*n* = 16)	OR (95% CI)	*p* value
Antacid use	140 (32%)	7 (43.8%)	1.66 (0.61–4.55)	0.319
Denture use	61 (14%)	6 (37.5%)	3.72 (1.30–10.60)	0.020^∗^
Smoking	58 (13.2%)	1 (6.3%)	0.44 (0.05–3.37)	0.706
Diabetes mellitus	49 (11.2%)	7 (43.8%)	6.19 (2.21–17.37)	0.001^∗^
Anemia	49 (11.2%)	4 (25%)	2.65 (0.82–8.54)	0.103
Helicobacter pylori	47 (10.7%)	1 (6.3%)	0.55 (0.07–4.31)	1.000
Corticosteroid use	30 (6.8%)	4 (25%)	4.54 (1.38–14.95)	0.025^∗^
Antibiotic use	27 (6.2%)	4 (25%)	5.08 (1.53–16.82)	0.018^∗^
Addiction	26 (6%)	1 (6.3%)	1.06 (0.13–8.33)	1.000
Cancer	23 (5.2%)	2 (12.5%)	2.58 (0.55–12.05)	0.217
Herbal medicine use	16 (3.6%)	1 (6.3%)	1.76 (0.22–14.18)	0.462
Alcohol use	9 (2%)	0 (0%)	NE	1.000
Organ transplantation	8 (1.8%)	0 (0%)	NE	1.000

*Note:* OR, univariate odds ratio; CI, 95% confidence interval. *p*‐values were calculated using Pearson’s chi‐square test; Fisher’s exact test was used when expected cell counts were small, as appropriate. NE, odds ratio not estimable because no cases occurred in the *Candida esophagitis* group.

^∗^Statistically significant at *p* < 0.05.

### 3.3. Distribution of *Candida* Species

**FIGURE 2:**
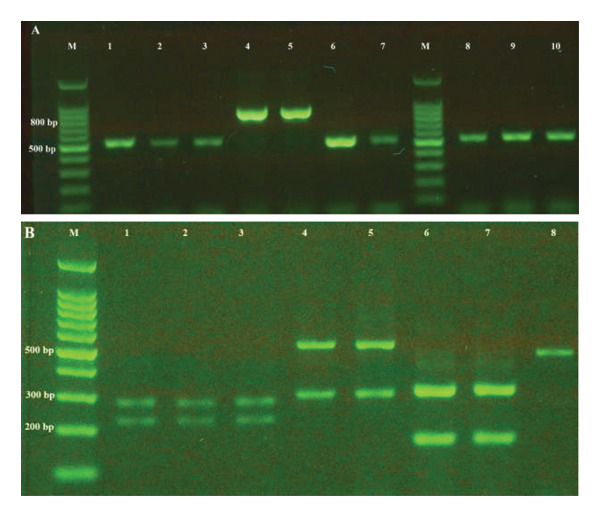
(A) PCR products of Candida species: lane M: 100‐bp DNA ladder; lanes 1–3 and 8–10, *C. albicans* (537 bp); lanes 4‐5, *C. glabrata* (881 bp); lane 6, *C. tropicalis* (526 bp); lane 7, *C. parapsilosis* (530 bp). (B) Agarose gel electrophoresis of RFLP products: lane M: 100‐bp DNA ladder; lanes 1–3, *C. albicans* (239, 298 bp); lanes 4‐5, *C. glabrata* (320, 561 bp); lanes 6‐7, *C. tropicalis* (186, 340 bp); lane 8, *C. parapsilosis* (530 bp).

Molecular analysis using PCR‐RFLP revealed that among 84 patients with positive *Candida* cultures, 73 (87%) were *C. albicans* and 11 (13%) were non‐*albicans* species, including *C. glabrata* (*n* = 7; 8.3%), *C. tropicalis* (*n* = 3; 3.6%), and *C. parapsilosis* (*n* = 1; 1.2%) (Figure 2). Among the 16 patients with CE, the predominant species was *C. albicans* (*n* = 13; 81.3%), followed by *C. glabrata* (*n* = 2; 12.5%) and *C. tropicalis* (*n* = 1; 6.2%).

## 4. Discussion

Most studies of CE have included patients with HIV although it can also be observed in patients with other predisposing factors [18, 19]. We performed a cross‐sectional study to assess the prevalence, clinical manifestations, risk factors, and *Candida* species distribution in patients with CE. *Candida* colonization occurs in approximately 20% of the healthy adults [20], and colonization represents a necessary step for fungal invasion of the epithelial layer in patients with immunodeficiency or other predisposing conditions [21, 22].

In our study, the prevalence of CE among HIV‐negative patients who underwent upper gastrointestinal endoscopy in whom esophageal biopsy was performed based on endoscopic findings (*n* = 455) was 3.5%, with diagnosis confirmed by endoscopy, histopathology, and culture. This prevalence is consistent with previous reports in immunocompetent populations, which range from 0.3% to 5.2% [4, 23, 24]. However, compared to a large South Korea study that evaluated over 88,000 predominantly younger healthy individuals and reported a CE prevalence of approximately 0.32% [4], the higher prevalence observed in our study may be partly due to the smaller sample size, as well as differences in demographic characteristics, regional variations, dietary habits, and comorbid conditions such as diabetes mellitus. These findings indicate that, while CE is primarily considered an opportunistic infection in immunocompromised individuals, it can also occur in patients with other predisposing factors, including metabolic conditions such as diabetes mellitus [25, 26].

In our study, the most common clinical symptoms among CE patients were epigastric pain (68.8%) and acid reflux (56.3%). These findings are consistent with previous studies. Choi et al. in South Korea reported acid reflux, epigastric pain, and dysphagia as the most frequent symptoms [4] and Takahashi et al. in Japan dysphagia, epigastric pain, and acid reflux to be common [24]. A study in Pakistan also reported retrosternal discomfort, dysphagia, and epigastric pain as common symptoms in non‐HIV patients [18]. Dysphagia was less frequent in our patients, which may be due to differences in demographics, clinical settings, or the small sample size.

Our study showed that diabetes mellitus (*p* = 0.001), denture use (*p* = 0.020), recent antibiotic use (*p* = 0.018), and corticosteroid use (*p* = 0.025) were significantly associated with CE. All CE patients had at least one of these identified predisposing factors; notably, none reported a history of alcohol use or organ transplantation. This highlights the role of these factors in the development of CE. Unlike previous studies, antacid use was not statistically associated with CE (*p* = 0.319). Patients with diabetes mellitus are more susceptible to fungal infections due to impaired phagocytic function, reduced chemotactic activity, and elevated blood and tissue glucose, which favor *Candida* growth [21]. Shad et al. showed that steroid therapy, diabetes, antibiotic use, and cancer are key risk factors for CE [27]. Similarly, Yakoob et al., in Pakistan, identified carcinoma, diabetes mellitus, corticosteroid use, and antibiotic therapy as major risk factors for CE [18]. Our finding of an association between use of dentures and CE aligns with the results of Loster et al., reporting that esophageal candidiasis was significantly more prevalent in patients with confirmed oral mucosal candidiasis compared to those without the condition. Therefore, the presence of oral fungal infection may act as a predisposing factor for the development of CE, emphasizing the importance of simultaneous diagnosis and treatment of both oral and esophageal infections [28]. Broad‐spectrum antibiotics can disturb normal bacterial flora, while corticosteroids suppress immune responses, both facilitating *Candida* overgrowth [21].

PCR‐RFLP molecular analysis revealed that among CE cases (*n* = 16), *C. albicans* was the predominant species (81.3%), followed by *C. glabrata* (12.5%) and *C. tropicalis* (6.2%). This distribution is consistent with previous reports, in which *C. albicans* accounts for 50%–70% of CE patients [29, 30]. In the study by Jafarian et al. on patients with CE, *C. albicans* was identified as the predominant species, followed by *C. glabrata* and *C. tropicalis* as the non‐*albicans* species [31]. Due to differences in antifungal susceptibility among *Candida* species and evidence from clinical and epidemiological studies, accurate identification of clinical *Candida* isolates using PCR‐RFLP is recommended.

In our study, PCR‐RFLP was performed solely for accurate identification of *Candida* species and for epidemiological purposes. Accordingly, we did not assess antifungal susceptibility, and no analyses were performed to evaluate differences in clinical severity or treatment response among the various *Candida* species. Future studies could incorporate antifungal susceptibility testing to better clarify the potential clinical implications of different species. Another limitation is that despite excluding patients with a documented history of recent antifungal use, information regarding prior antifungal exposure was obtained through self‐reported data collected via the study questionnaire. While no participant reported using systemic or topical antifungal agents prior to the endoscopy, self‐reported medication history may be subject to recall or reporting bias, which could potentially influence culture outcomes. Finally, the single‐center design and the relatively small number of active CE cases limit the generalizability of our findings, highlighting the need for larger, multicenter investigations to confirm these results.

## 5. Conclusion

In summary, diabetes mellitus, denture use, recent antibiotic therapy, and corticosteroid use were significant risk factors for CE in HIV‐negative patients, and *C. albicans* was identified as the predominant species. These findings highlight the importance of early identification and management of predisposing factors to prevent recurrence of CE.

## Author Contributions

Fatemeh Nabiei and Milad Badri: sample collection; Rasoul Samimi: endoscopic procedures; Neda Nasirian: histopathological examinations; Monirsadat Mirzadeh: statistical analysis; Faezeh Mohammadi: study design and supervision. All authors contributed to writing of the final version of the paper.

## Funding

This research was supported financially by the Research Deputy of Qazvin University of Medical Sciences, Iran (contract no. IR.QUMS.REC.1402.100).

## Ethics Statement

The Ethics Committee of Qazvin University of Medical Sciences, Iran (IR.QUMS.REC.1402.100), approved this study. Written informed consent was obtained from all participants, and all procedures adhered to the ethical principles outlined in the Declaration of Helsinki.

## Conflicts of Interest

The authors declare no conflicts of interest.

## Data Availability

The data that support the findings of this study are available from the corresponding author upon reasonable request.
